# Analysis of pediatric emergency department patient volume trends during the COVID-19 pandemic

**DOI:** 10.1097/MD.0000000000026583

**Published:** 2021-07-09

**Authors:** Matthew Philip Pepper, Ernest Leva, Prerna Trivedy, James Luckey, Mark Douglas Baker

**Affiliations:** Pediatrics, Rutgers Robert Wood Johnson Medical School, New Brunswick, NJ.

**Keywords:** COVID-19, patient census, patient volume

## Abstract

During the early period of the COVID-19 pandemic there was a substantial decrease in pediatric emergency department (PED) visitation. The intent of this study is to report PED utilization during the COVID-19 pandemic in an urban pediatric referral center located close to the epicenter in the northeastern US.

A retrospective analysis of medical records of patients visiting the PED at Robert Wood Johnson University Hospital (RWJUH) was performed. Data included: daily census, admission rate, Emergency Severity Index, and ICD-10 diagnosis codes for the period of February through July, 2018 to 2020.

By the week of March 26th, visits had decreased by 70% compared to the average of the previous 2 years. This census nadir lasted for 6 weeks. At 5 weeks postnadir the average daily census recovered to levels 40% lower than prior year norms and remained at that level during subsequent months. The greatest decreases were seen in low-acuity visits. Visits for behavioral health and fractures decreased by approximately 50% and 70%, respectively, but recovered to prior year norms by June and July of 2020. Visits for asthma exacerbation decreased by as much as 87% and remained at record lows for the remainder of the study period.

A substantial and persistent decrease in PED visitation was experienced during the COVID-19 pandemic. Whereas visits for behavioral health and fractures have recovered to prior year norms, visits for asthma exacerbation remain at record lows. Further research is needed to ascertain the causes of these changes, including patient perceptions of the PED.

## 1 Introduction

The early period of the COVID-19 pandemic resulted in a large decrease in emergency department (ED) visitation. A 42% reduction in US ED visits was reported in the period from March 29 through April 25, 2020 when compared with the prior year.^[1]^ Similar trends were identified in Italy, Spain, Norway, and Turkey.^[2–7]^ These decreases were most pronounced in the pediatric population, with 1 report finding a 71% decrease in US ED visits for patients ≤14 years old.^[1]^ The Northeast region of the US saw sharper declines than other areas. Visits for children ≤10 years old decreased substantially for common infectious and respiratory diagnoses, including a 97% decrease in visits for influenza, an 85% decrease in visits for otitis media, and an 84% reduction in visits for asthma.

It has been theorized that cancellation of daycare, schools, organized sports, and other multi-person activities led to a reduction of transmission of infectious diseases and occurrence of traumatic injuries in the pediatric population.^[5]^ It has also been conjectured that parents and patients became hesitant to seek care in the ED due to fear of becoming infected with SARS-CoV-2.

The pediatric emergency department (PED) at Robert Wood Johnson University Hospital (RWJUH) is a full-service 15 bed facility situated on the same campus as Bristol Myers Squibb Children's Hospital and the Child Health Institute of New Jersey, and provides emergency services for children who are cared for at these institutions and others. The PED typically serves 26,000 patients per year, ages 0 through 20 years. The PED is located in central New Jersey, flanked by New York City to the north, and Philadelphia to the south. The intent of this study is to report PED utilization during the COVID-19 pandemic in an urban pediatric referral center located close to the epicenter in the northeastern US.

## 2 Materials

A retrospective analysis of medical records of patients visiting the PED at RWJUH was performed using the institution's electronic medical record, Emergency Department Information Management, E∗Healthline (Sacramento, CA). The data collected included: daily census, daily admission rate, triage Emergency Severity Index, and ICD-10 diagnosis codes for the period of February through July for each of 3 successive years, 2018 to 2020. Diagnostic codes for asthma exacerbation (ICD-10 J45.901, J45.902, J45.909), skeletal fractures, and mental/behavioral health visits (see appendix) were selected using Microsoft Excel. Graphs were created in Microsoft Excel. This study received approval from the Rutgers University Institutional Review Board. The datasets generated during and/or analyzed during the current study are available from the corresponding author on reasonable request.

## 3 Results

**Figure 1:**
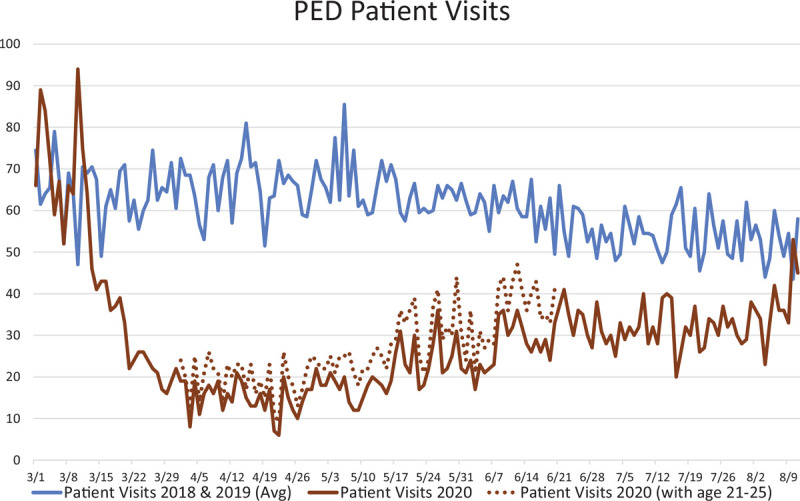
PED patient visits.

The index case of COVID-19 on our campus was an adult patient who was admitted to RWJUH on March 14, 2020. In the days following the March 17th closure of schools in NJ, there was a substantial decrease in PED patient visits that continued for 3 weeks, until a low steady state was established (Fig. 1). By the week of March 26th, visits had decreased by at least 70% when compared to the average of the previous 2 years. This census nadir lasted for 6 weeks (April 4 through May 16, 2020) before beginning to gradually increase. The average daily census during the 2020 nadir was 15.8 vs 66.2 for the same time interval during the prior 2 years. At 5 weeks postnadir (June 20, 2020), the average daily census recovered to levels approximately 40% lower than prior year norms and remained at that volume during subsequent summer months.

**Figure 2:**
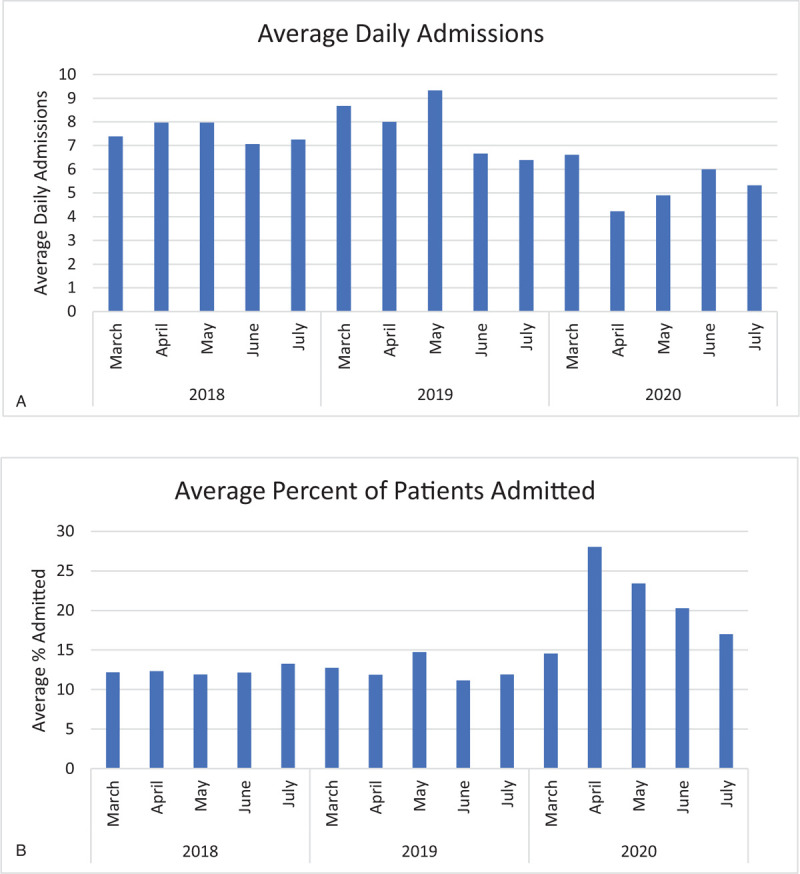
(A). Average daily admissions. (B). Average percent of patients admitted.

Compared to prior years, fewer children were admitted from the PED to the in-patient services during the COVID-19 pandemic. However, the percentage reduction was less pronounced than the reduction in overall visits to the PED. During the PED census nadir (April 4 through May 16, 2020), the average daily in-patient admissions dropped to 4.2 per day, representing a 49% reduction from in-patient admissions during the same time periods in 2018 and 2019 (Fig. 2A). Like the overall PED census, in-patient admission numbers increased gradually postnadir, recovering to levels 20% lower than prior year norms. Between April 1st and May 31st in 2018 and 2019, an average of 12% of patients seen in the ED were admitted to the hospital, whereas an average of 23% of patients evaluated in the ED were admitted to the hospital during that time period in 2020 (Fig. 2B).

Starting on April 1st, to assist the adult ED, the maximum age of patients seen in the PED was increased from age 20 to 25 (Fig. 1). These patients accounted for an average of 23% of patients seen in the PED and were not included in this analysis.

**Figure 3:**
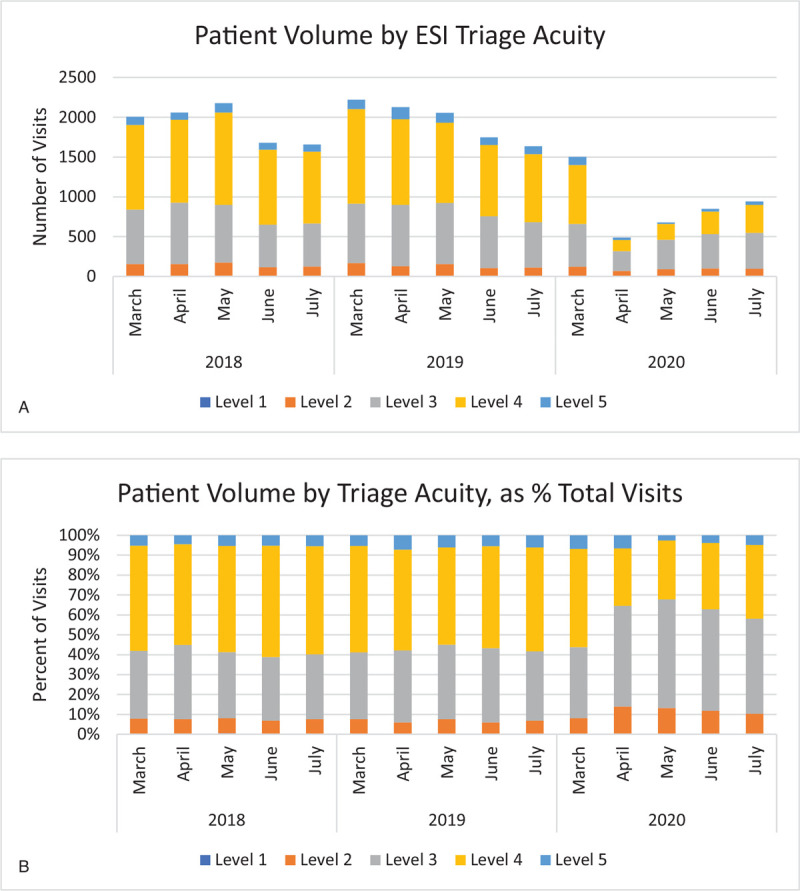
(A). Patient volume by ESI triage acuity. (B). Patient volume by ESI triage acuity, as % total visits. ESI = Emergency Severity Index.

In April and May of 2018 and 2019, triage level 2, 3, and 4 visits accounted for an average of 7%, 36%, and 51% of all patient visits, respectively (Fig. 3B). Emergency Severity Index triage levels range from 1 (most urgent) to 5 (least urgent). In the same months of 2020, visits of these triage levels accounted for 13%, 52%, and 29% of all visits. In absolute terms, Triage level 2 visits decreased by 48% in 2020 when compared with previous years, whereas Triage level 3 and 4 visits decreased by 59% and 84%, respectively (Fig. 3A).

**Figure 4:**
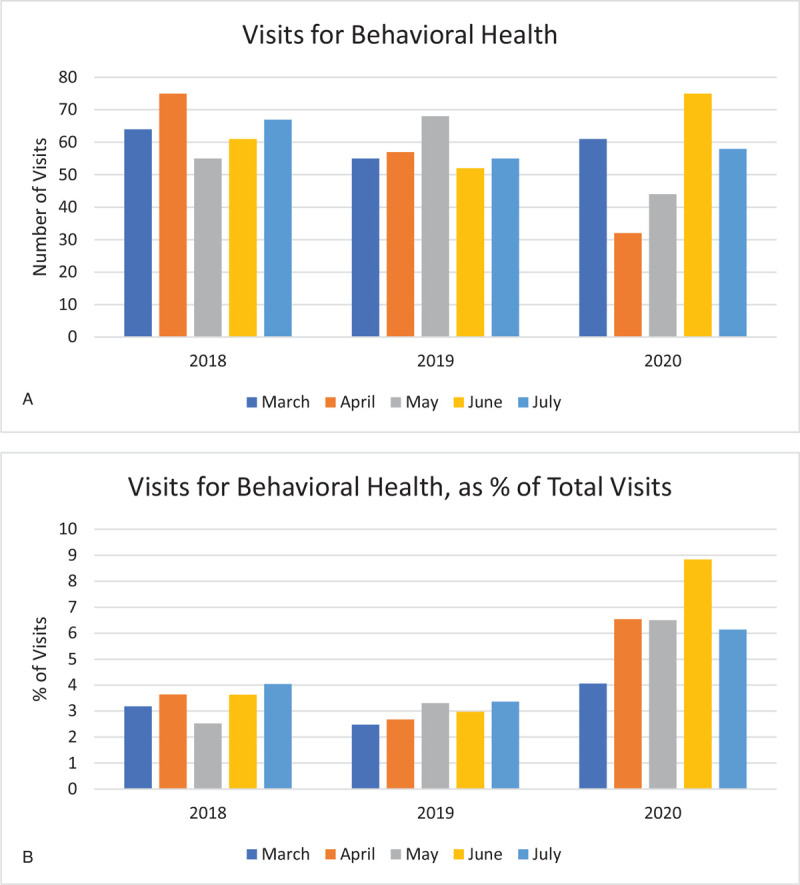
(A). Visits for behavioral health. (B). Visits for behavioral health, as % of total visits.

Visits for behavioral health decreased from an average of 66 per month in April 2018 and 2019 to 32 per month in 2020 (Fig. 4A) (see Appendix, Supplemental Content, which lists diagnoses included in analysis). As a percentage of total ED visits this amounted to an increase from 3.2% in previous years to 6.5% in 2020 (Fig. 4B). During subsequent months in 2020, behavioral health visits gradually increased, approximating prior year norms by June.

**Figure 5:**
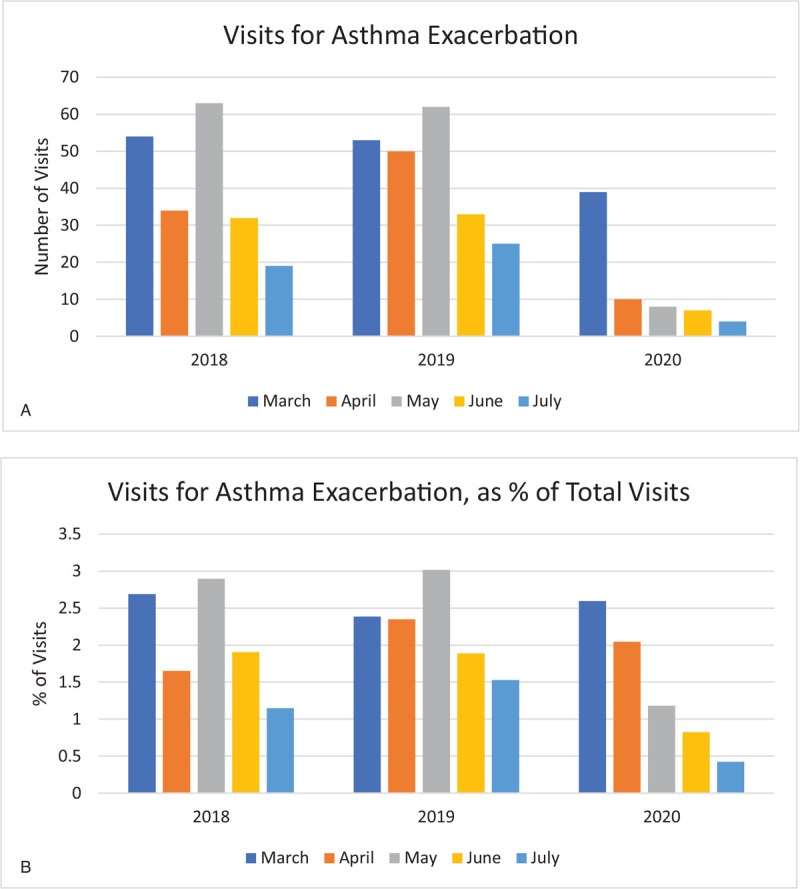
(A). Visits for asthma exacerbation. (B). Visits for asthma exacerbation, as % of total visits.

Visits for asthma exacerbation in April decreased from an average of 44 per month in previous years to 10 in April 2020 (Fig. 5A). As a percentage of visits, asthma exacerbation accounted for 2% of visits both in April of previous years and in April 2020 (Fig. 5B). In May, there were only 8 patient visits for asthma exacerbation, accounting for 1.2% of visits, compared to the 62 visits seen in previous years, which accounted for 3% of visits. Visits for asthma exacerbation continued to experience further decreases, with only 7 visits in June and 4 in July.

**Figure 6:**
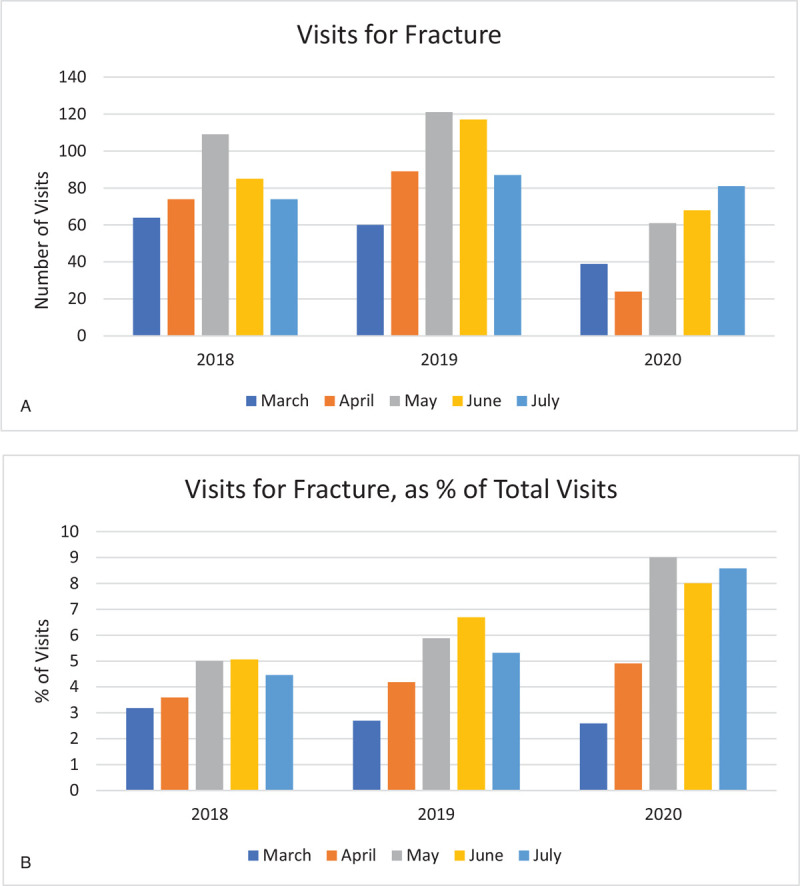
(A). Visits for fracture. (B). Visits for fracture, as % of total visits.

Visits for fractures increased from March through May, then declined during summer months in 2018 and 2019 (Fig. 6A). However, in 2020, there was a sharp decline in numbers of fractures managed during the COVID-19 surge in April, after which there was a gradual month-to-month increase, reaching pre-COVID-19 experience in July. When expressed as a percentage of total visits, the month-to-month increase related to fractures was more pronounced in 2020 than in previous years (Fig. 6B).

## 4 Discussion

In our urban PED, we experienced a substantial and prolonged decrease in patient volume during the COVID-19 pandemic. This is probably due to more than 1 cause. Infectious complaints typically account for 27%, and traumatic injuries account for 11% of PED visits nationwide among Medicaid patients.^[8]^ The closure of daycare facilities and schools, along with widespread social distancing undertaken to prevent SARS-CoV-2 transmission, deceased opportunities for transmission of more common infectious diseases. Likewise, the cancellation of organized sports and other interactive physical activities likely decreased the number of patients with minor traumatic injuries that would usually be evaluated in the PED. Resumption of those activities during summer months paralleled the subsequent increase fracture-associated visits. The location of our PED next to the New Brunswick Campus of Rutgers University also played a role in the decrease in patient volume. The campus has over 50,000 enrolled students during a typical school year, many of whom live in other areas of the state or country when classes are not in session. When classes were canceled on March 12, 2020, most students left the area, no longer seeking medical care at our facilities.^[9]^

It is evident that many parents and patients with minor illnesses avoided using the PED due to fear of contracting SARS-CoV-2 during their visit. Patients might also have avoided the hospital due to the expectation of overcrowding and long wait times, as is often reported in the popular press regarding adult EDs in the New York metro area. Indeed, as reported in the lay press, utilization of public transportation remained substantially decreased from normal throughout the study period.^[10]^ As a result of the COVID-19 pandemic, there have been widespread efforts to increase telemedicine and phone triage services in all clinical practice settings.^[11]^ Patients are contacting their primary medical doctors to address acute issues by virtual means, rather than visit emergency departments for evaluation. Many patients are also using commercial telemedicine services, with 1 study reporting a 154% increase in telehealth visits during the early pandemic.^[12]^ Our institution's outpatient pediatric practices initiated the use of telemedicine during the study period, potentially contributing to decreased PED patient visitation.

Poor air quality contributes to exacerbations of asthma, cystic fibrosis, and respiratory infections in children.^[13]^ Contributing to air pollution are the components of vehicular exhaust, including fine particulate matter (PM2.5), Nitrogen Dioxide (NO2), and Nitrogen Oxides (NOX).^[14]^ Changes in air quality related to the COVID-19 pandemic have been studied in Milan, Italy, which enacted a partial lockdown on March 23rd, resulting in drastic reductions in air pollution.^[15]^ PM2.5 decreased by approximately 37%, NO2 by 43%, and NOx by 59.9%. Levels further decreased when a total lockdown was enacted. These decreases in pollution have been attributed to decreased vehicular traffic (estimated as a 75% reduction), reduced industrial activity, and reduced heating of offices and other workplaces. New Jersey experienced an estimated 61% decrease in vehicular traffic on major roadways as of April 2020.^[16]^ In Newark, New Jersey, during the month of March 2020, the hourly average level of PM2.5 decreased by 27%, NO2 by 34%, and NOX by 46%.^[17]^ Levels of NO2 and NOX further decreased during the month of April, hourly averages of these gasses declining by 57% and 65%, respectively, when compared with the first weeks of March 2020. We suspect that these improvements in air quality resulted in fewer asthma exacerbations and other respiratory illnesses.

Decreased vehicular traffic also results in fewer automobile collisions and fewer resultant traumatic injury visits to the PED. California, which implemented stay at home orders at the start of the COVID-19 pandemic, experienced an estimated 55% reduction in vehicular traffic and a 50% reduction in collisions resulting in injury.^[18]^ In New Jersey, there was a 35% decrease in fatal vehicular accidents in April 2020 when compared to the previous 5 years, the lowest level in the last 50 years.^[16]^

Changes to the economy are also possible causes of decreased patient volume in the PED. As of May 2020, New Jersey had a 15.2% unemployment rate.^[19]^ Unemployment and financial hardship may lead many patients to avoid seeking medical care due to interrupted insurance coverage and concerns about inability to pay for their care. Furthermore, economic hardships caused by the pandemic have disproportionately affected some sectors of the population. As of July 2020, unemployment rates were higher for Latinos, who comprise 50% of New Brunswick residents.^[20,21]^ Conversely, widespread unemployment and the loss of employee-sponsored insurance may eventually lead patients to rely on the ED for medical care, as occurred during the 2008 economic downturn.^[22]^

It is important to note that the pandemic had a less pronounced effect on the utilization of the PED for evaluation and management of behavioral health issues and fractures than it did for asthma exacerbations. It would seem that the changes in human behavior that came about during the surge of COVID-19 in the northeast US more substantially affected the occurrence of respiratory illnesses like asthma, and had lesser impact on the occurrences of behavioral health illnesses and physical trauma, which are less dependent on human-to-human interactions.

This study is limited by the nature of the data collected in that it is not possible to determine the causes of the identified changes in patient volume. While it is strongly suspected that changes in patient volume were caused by reduced transmission of common infectious illnesses, decreased traumatic injuries, avoidance of the PED, and the use of telemedicine, it is not possible to determine the roles, if any, that these factors played without further study.

## 5 Conclusion

The COVID-19 pandemic has led to a considerable decrease in PED visits. The greatest decreases were seen in low-acuity visits. While visits related to behavioral health have recovered to their baseline frequency, visits for asthma exacerbation remain at record lows. These changes were caused by multiple factors, and further research is needed to elucidate the roles of air pollution, loss of health insurance, and patient perceptions of the PED.

## Author contributions

**Conceptualization:** Matthew Philip Pepper, Mark Douglas Baker, Ernest Leva.

**Data curation:** Matthew Philip Pepper, Prerna Trivedy, James Luckey.

**Formal analysis:** Matthew Philip Pepper, James Luckey.

**Investigation:** Matthew Philip Pepper, Prerna Trivedy, James Luckey.

**Methodology:** Matthew Philip Pepper, Mark Douglas Baker.

**Project administration:** Matthew Philip Pepper, Mark Douglas Baker, Ernest Leva.

**Supervision:** Mark Douglas Baker, Ernest Leva.

**Visualization:** Matthew Philip Pepper, Prerna Trivedy.

**Writing – original draft:** Matthew Philip Pepper.

**Writing – review & editing:** Matthew Philip Pepper, Mark Douglas Baker, Ernest Leva, Prerna Trivedy, James Luckey.

## Supplementary Material

Supplemental Digital Content
