# Supplementary material for: Analysis of pediatric emergency department patient volume trends during the COVID-19 pandemic
Source: Medicine (Baltimore). 2021 Jul 9;100(27):e26583. doi: 10.1097/MD.0000000000026583 (PMC8270607; doi:10.1097/MD.0000000000026583)
Supplement: Supplemental Digital Content [file medi-100-e26583-s001.docx]

**Appendix**

**ICD -10 Mental Health Codes**

**Adjustment disorders**

ICD-10: F43.20-F43.9

**Anxiety disorders**

ICD-10: F06.4, F40.00-F40.11, F402.10-F402.98, F40.8-F40.9, F41.0-F41.9, F42-F42.9, F43.0-F43.12, F48.8-F48.9, R45.2-R45.84

**Delirium dementia and amnestic and other cognitive disorders**

ICD-10: F01.50-F01.51, F02.80-F02.81, F03.90-F03.91, F04, F05, F07.0-F07.9, F09, F48.2, G30.0-G30.9, G31.01-G31.83, R41.81, R54

**Developmental disorders**

ICD-10: F70-F79, F80.0-F80.9, F81.0-F81.9, F82-F89, F98.5, R41.83, R48.0

**Disorders usually diagnosed in infancy, childhood, or adolescence**

ICD-10: F64.2, F84.0-F84.9, F93.0-F93.9, F94.0-F94.9, F95.0-F95.9, F98.0-F98.9

**Mood disorders**

ICD-10: F06.30-F06.34, F30.10-F30.9, F31.0-F31.9, F32.0-F32.9, F33.0-F33.9, F34.0-F34.9, R45.86

**Personality disorders**

ICD-10: F60.0-F60.9, F69

**Schizophrenia and other psychotic disorders**

ICD-10: F06.0-F06.2, F20.0-F20.9, F21-F29

**Miscellaneous mental health disorders**

ICD-10: F06.1-F06.8, F44.0-F44.9, F45.0-F45.9, F48.1, F50.00-F50.9, F51.01-F51.9, F52.0-F52.9, F53-F59, F64.0-F64.9, F65.0-F65.9, F66, F68.10-F68.8, F99, O90.6, R37, R45.89, Z87.890, Z91.83

**Attention-deficit conduct and disruptive behavior disorders**

ICD-10: F90.0-F90.9, F91.0-F91.9, R46.0-R46.89

**Impulse control disorders NEC**

ICD-10: F63.0-F63.9, R458.50

**Alcohol-related disorders**

ICD-10: F101.0-F101.9, F102.0-F102.59, F10.26-F10.27, F102.80-F102.9, F109.20-F109.9, G62.1, I42.6, K292.0-K292.1, K70.0-K70.9, O99.310-O99.315, P04.3, Q86.0

**Substance-related disorders**

ICD-10: F111.0-F111.9, F112.0-F112.9, F119.0-F119.9, F121.0-F121.9, F122.0-F122.9, F129.0-F129.9, F131.0-F131.9, F132.0-F132.9, F139.0-F139.9, F141.0-F142.1,

F142.20-F142.9, F149.0-F149.9, F151.0-F151.9, F152.0-F152.9, F159.0-F159.9, F161.0-F161.9, F162.0-F162.9, F169.0-F169.9, F172.00-F172.99, F181.0-F181.9,

F182.0-F182.9, F189.0-F189.9, F191.0-F191.9, F192.0-F192.9, F199.0-F199.9, F55.0-F55.8, O35.5XX0-O35.5XX9, O99.320-O99.325, P04.41-P04.49, P96.1-P96.2,

T40.0X1A-T40.996S

**Suicide and intentional self-inflicted injury**

ICD-10: R45.851, T14.91-T14.91XS, T36.0X2A-T50.Z92S, T51.0X2A-T65.92XS, T71.112A-T71.232S, X71.0XXA-X83.8XXS, Z91.5

**Screening and history of mental health and substance abuse**

ICD-10: R78.0-R78.6, Z04.6, Z13.4, Z72.810-Z72.811, Z86.51-Z86.59, Z87.891, Z91.410-Z91.49
